# RAA-CRISPR/Cas12a-driven two-tube, one-tube and one-tube-LFS for rapid detection of feline parvovirus

**DOI:** 10.3389/fvets.2025.1707332

**Published:** 2025-12-12

**Authors:** Guangyu Ge, Zhiyi Chen, Siyuan Liu, Zhonglin Tang, Yulong Yin, Qinghua He

**Affiliations:** 1 Department of Food Science and Engineering, College of Chemistry and Environmental Engineering, Shenzhen University, Shenzhen, Guangdong, China; 2 Shenzhen Branch, Guangdong Laboratory for Lingnan Modern Agriculture, Genome Analysis Laboratory of the Ministry of Agriculture, Agricultural Genomics Institute at Shenzhen, Chinese Academy of Agricultural Sciences, Shenzhen, Guangdong, China; 3 Laboratory of Animal Nutritional Physiology and Metabolic Process, Institute of Subtropical Agriculture, The Chinese Academy of Sciences, Changsha, Hunan, China; 4 Shenzhen Key Laboratory of Food Macromolecules Science and Processing, Shenzhen University, Shenzhen, China

**Keywords:** RAA, CRISPR/Cas12a, Two-tube, One-tube, lateral flow strip

## Abstract

A One-tube method biosensor was developed based on Two-tube recombinase-aid amplification (RAA)-CRISPR/Cas12a system due to the risk of aerosol contamination caused by open-tube operations in the Two-tube method. To further enable naked-eye visualization and portable detection of feline parvovirus (FPV), the lateral flow strip (LFS) technology was introduced to construct One-tube-LFS method. The limits of detection (LODs) of the One-tube and Two-tube methods were determined to be both 4.277 copies/μl, while the One-tube-LFS method was 42.77 copies/μl, which exhibited an LOD comparable to quantitative real-time polymerase chain reaction (qPCR). Notably, no cross-reactivity was observed with other common feline pathogens and the consistency of the three methods with qPCR results ranged from 97.22 to 100% in applications of 36 clinical samples. These findings demonstrated that One-tube, two-tube and One-tube-LFS biosensors were capable of rapidly, sensitively, and specifically detecting FPV. The RAA and CRISPR/Cas12a systems were spatially segregated, with the former placed at the bottom and the latter at the cap of the tube. This strategy effectively avoided the cleavage of target DNA and primers by Cas12a during the critical exponential amplification phase of RAA, thereby significantly enhancing the DNA amplification efficiency. The three biosensors could be used for on-site detection in 1 h, and the results could be visualized through fluorescence quenching or LFS. These techniques provide point-of-care testing solutions for clinical diagnosis and rapid screening, especially in resource-limited settings.

## Graphical Abstract

**Figure:**
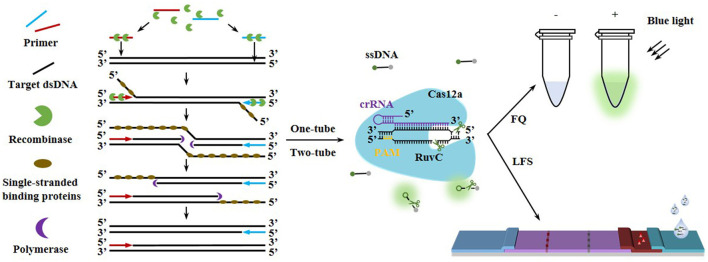
Fluorescence quenching (FQ) and lateral flow strip (LFS) detection schematics via RAA-CRISPR/Cas12a.

## 1 Introduction

In the diagnosis of animal viral infections, the preliminary diagnostic was based on the clinical symptoms and pathological changes of the infected animals. For further confirmation, methods such as virus isolation (1), hemagglutination inhibition test (2), and next-generation sequencing analysis (3) were required. These methods were considered stable and reliable, but they must be conducted in specialized laboratories, which presented complexities and time-consuming drawbacks. Although PCR (4) and real-time fluorescence quantitative PCR (qPCR) (3), which were regarded as the gold standards for molecular detection, offered high sensitivity advantages, the sample preparation and detection of viral nucleic acids still required specific working conditions and professional personnel. Obviously, they were not suitable for the deployment of point-of-care testing (POCT) protocols in resource-limited areas. Colloidal gold immunochromatography (5) and enzyme-linked immunosorbent assay (2) were applicable for POCT due to they did not require expensive equipment and complex amplification processes. However, they relied on sensitive, easily degradable reagents, and their low detection sensitivity often resulted in false-negative outcomes, which could not accurately exclude infections. In contrast, isothermal amplification techniques, characterized by low instrument dependency and high nucleic acid amplification efficiency, were more suitable for POCT, including strand displacement amplification, loop-mediated isothermal amplification, rolling circle amplification, and recombinase polymerase amplification/recombinase-aided amplification (RPA/RAA) (6–8). Among them, RPA/RAA was deemed the most effective and promising isothermal amplification technology due to its rapidity, high sensitivity, and moderate reaction temperature (9). It was evident that combining RPA/RAA with agarose gel electrophoresis did not significantly improve upon PCR methods. A recent study demonstrated that the combination of RPA with lateral flow dipsticks (RPA-LFD) exhibited outstanding performance in terms of time, cost, and efficiency, making it a promising candidate for on-site detection of feline parvovirus (FPV) (10). However, LFDs were unable to detect asymptomatic carriers with low viral titers, potentially leading to false-negative results. Moreover, the opening of tubes during the incubation process of LFDs could easily cause aerosol pollution of RPA products.

In recent years, the Clustered Regularly Interspaced Short Palindromic Repeats/CRISPR-associated (CRISPR/Cas) system had experienced rapid development due to its high sensitivity, speed, and ease of use, particularly the Cas12 and Cas13 systems. These class II of RNA-guided Cas proteins had demonstrated broad prospects in various nucleic acid diagnostic applications (11–13). They initially bound specifically to target genes and subsequently activated the Cas enzymes through guide RNA, which triggered the trans-cleavage activity of the Cas enzymes to randomly degrade non-target strands, thereby generating fluorescent signals for qualitative or quantitative detection of target genes. In this context, the combination of isothermal amplification technology and the CRISPR/Cas system had emerged as a new trend in nucleic acid detection due to its exceptional sensitivity, and had significantly advanced in the detection of bacteria, viruses, fungi, parasites, toxins, and other chemical contaminants (11, 13, 14). Among animal viruses, the integration of isothermal amplification technology with the CRISPR/Cas system had been demonstrated to be effective for the detection of African swine fever virus (15), porcine circovirus (16), feline herpesvirus type 1 (17), monkeypox virus (18), and avian influenza virus (19). However, compared to other animal viruses, the development of FPV diagnostic methods based on the CRISPR/Cas system had been relatively lagging. Recently, Two-tube method based on RPA and CRISPR/Cas12a technology (RPA-CRISPR/Cas12a) was first employed for the detection of FPV. The results indicated that the RPA-Cas12a system exhibited higher sensitivity and specificity, and notably, test results could be read using a simple handheld UV lamp in resource-limited areas (20). Similarly, sensor assay was developed to employ the transfer of RAA products into the CRISPR/Cas12a system to activate Cas12a-mediated cleavage for the detection of FPV (21). Due to the high sensitivity of the method, the opening of tubes after RPA amplification was highly vulnerable to environmental contamination by accumulated small-molecular nucleic acids, potentially leading to false-positive results. These not only increased the risk of aerosol contamination but also added operational steps. These drawbacks imposed more stringent requirements on the operation process, especially in primary-level detection.

**Figure 1:**
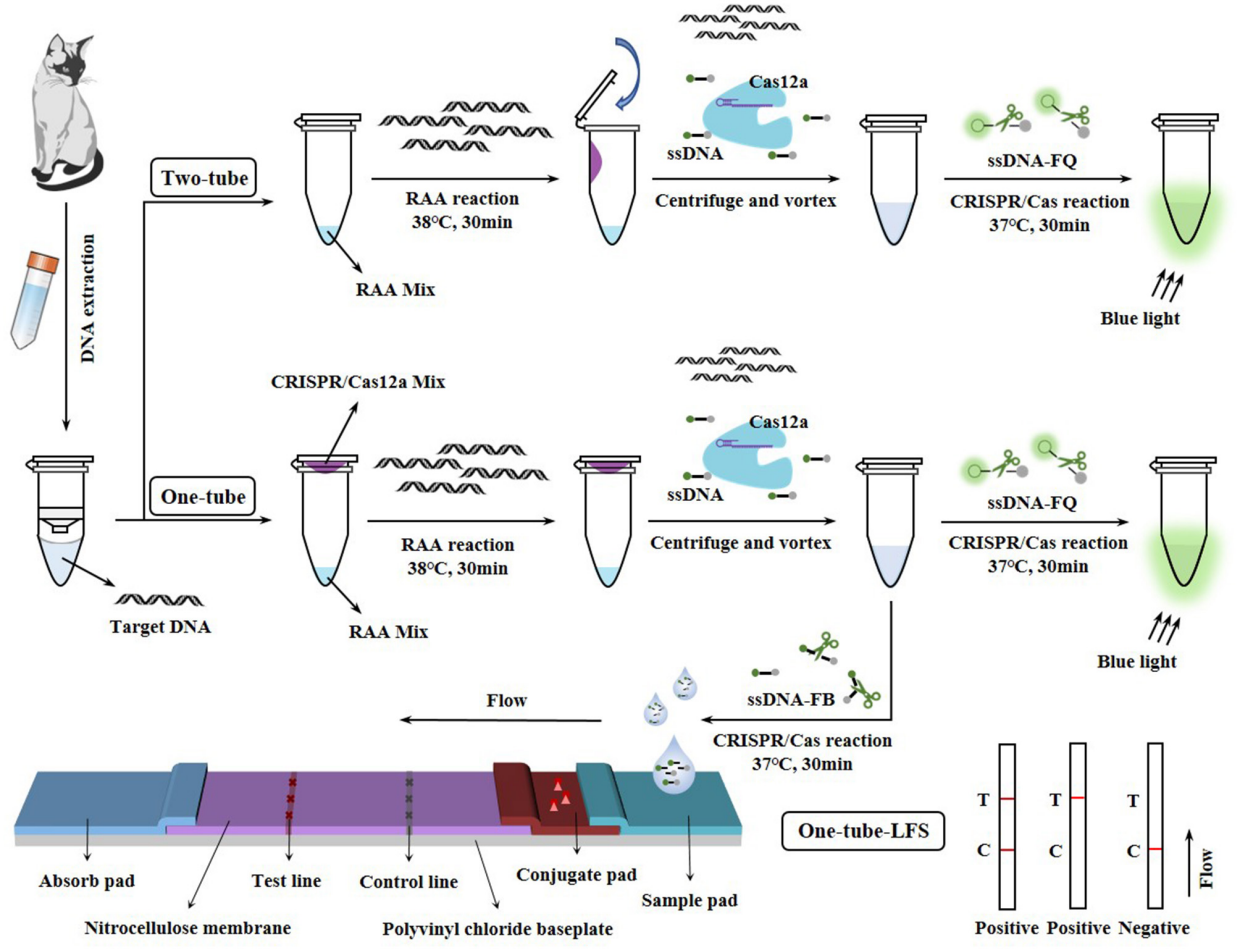
Two-tube, One-tube, and One-tube-LFS methods based on the RAA-CRISPR/Cas12a system.

FPV as one of the three major infectious pathogens in cats, seriously affects the health of cats (2). In recent years, there have been increasing reports of outbreaks caused by FPV both at home and abroad (22–27). In order to further prevent the prevalence of FPV, it is urgent to establish a convenient, rapid, and accurate POCT method for FPV in domestic and wild animals. To meet these challenges, Two-tube, One-tube, and One-tube-LFS methods based on the RAA-CRISPR/Cas12a system were constructed in this study for the detection of FPV (Figure 1). Firstly, the RAA reaction was optimized to rapidly and accurately enrich the target DNA, and further optimization was performed in combination with the CRISPR/Cas12a system to obtain better fluorescence signals. Secondly, to effectively prevent contamination caused by tube opening, Two-tube and One-tube methods were developed by placing the RAA and CRISPR/Cas12a reactions at the bottom and cap of the tube, respectively, and One-tube-LFS method was established by integrating LFS technology, enabling the detection of generated fluorescence signals via a blue light instrument or test strips. Finally, these methods were applied to detect clinical samples and compared with the qPCR method. These three RAA-CRISPR/Cas12a diagnostic tools for FPV nucleic acid detection were expected to facilitate the early diagnosis, large-scale screening, and prevention of asymptomatic and subclinical FPV infections.

## 2 Results

### 2.1 RAA system

**Figure 2:**
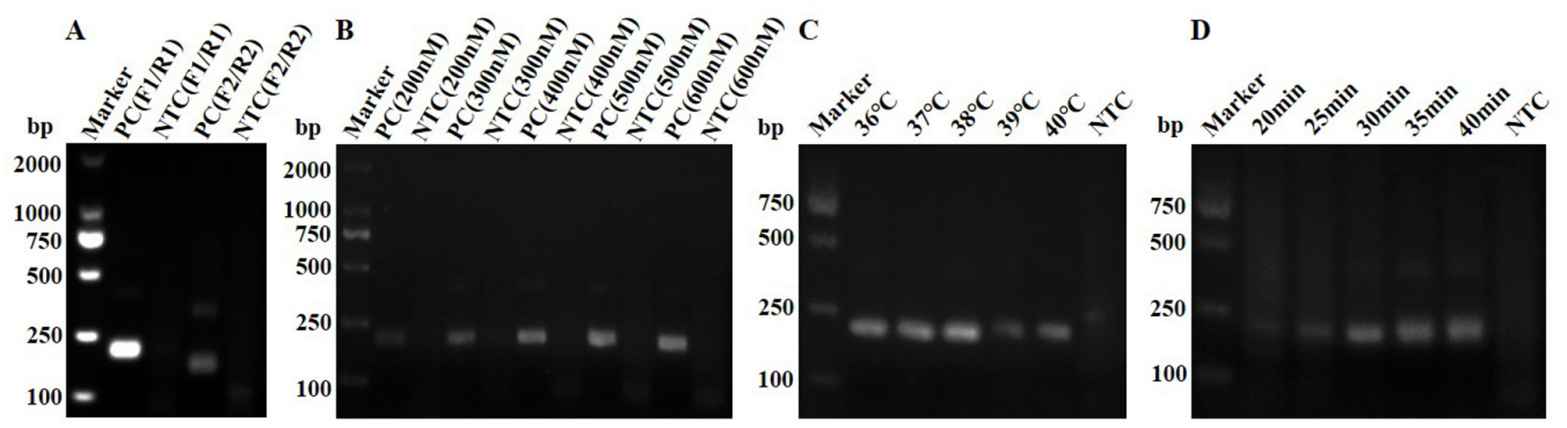
Optimization of the RAA system. **(A)** Primer screening for RAA. **(B)** Optimization of primer concentration for RAA. **(C)** Optimization of reaction temperature for RAA. **(D)** Optimization of reaction time for RAA.

Two pairs of primers were used for RAA amplification, and the results were analyzed by 2% agarose gel electrophoresis (Figure 2A). The primer pairs F1/R1 and F2/R2 appeared the target bands at about 194 and 159 bp, respectively. Among them, the band of primer pair F1/R1 was greater intensity, so the primer pair of F1/R1 was selected for subsequent experiments. Meanwhile, the optimized concentration of the F1/R1 primers was determined to be 500 nM (Figure 2B). The optimized RAA reaction temperature was 38 °C (Figure 2C), with a reaction time of 30 min (Figure 2D).

### 2.2 CRISPR/Cas12a system

**Figure 3:**
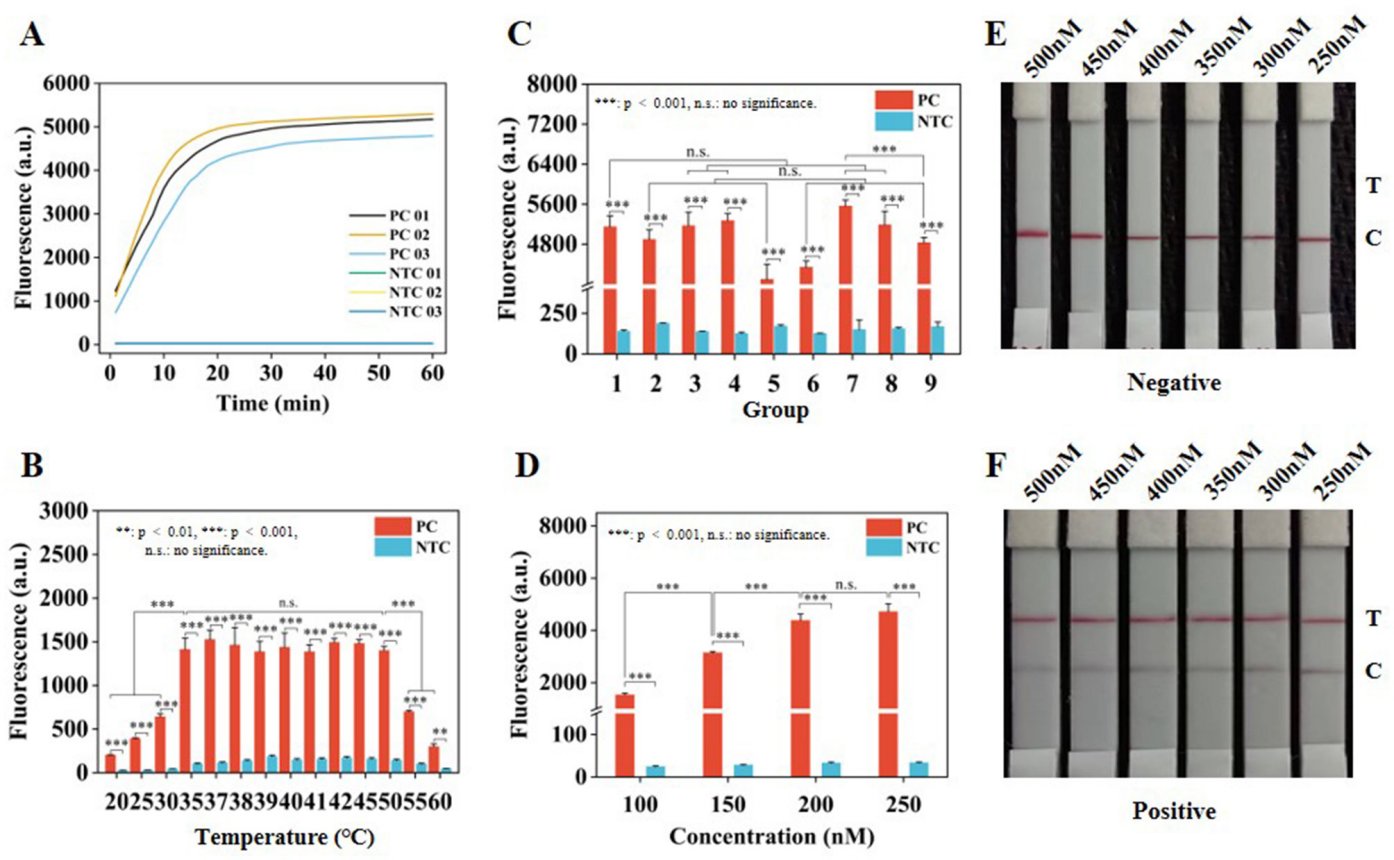
Optimization of CRISPR/Cas12a system. **(A)** Real-time fluorescence collection curves of crRNA in the three groups. **(B)** Fluorescence intensity at different temperatures for 30 min. **(C)** Fluorescence intensity in nine groups of complexes at 30 min. **(D)** Fluorescence intensity of ssDNA-FQ reporter at 30 min. **(E)** Optimization of ssDNA-FB concentration under negative conditions. **(F)** Optimize the concentration of ssDNA-FB under positive conditions. Error lines represent the mean ± S.D., of *n* = 3 replicates. **p < 0.01. ***p < 0.001. n. s., no significance.

Two crRNA sequences (crRNA01 and crRNA02) were designed and synthesized (Supplementary Table S1). Their effectiveness for fluorescence visualization was validated at concentrations of 4.277 × 10^10^, 4.277 × 10^9^, and 4.277 × 10^8^ copies/μl. Both crRNAs showed good fluorescence visualization results (Supplementary Figure S1A). Subsequently, three groups of crRNAs were established, including crRNA01, crRNA02, and crRNA03 (a mixture of crRNA01 and crRNA02 in equal proportions), designed to screen for the most effective crRNA and investigate whether a synergistic effect would be achieved by combining crRNA01 and crRNA02. Real-time fluorescence collection was performed at a plasmid concentration of 4.277 × 10^9^ copies/μl, allowing clear differentiation between the positive control (PC) and no template control (NTC) groups under blue light instrument (Supplementary Figure S1B). Among these, crRNA02 exhibited superior fluorescence detection performance compared to crRNA01 and crRNA03 (Figure 3A). Meanwhile, the fluorescence intensity was compared at 30 min of incubation, and crRNA02 also displayed a higher fluorescence intensity value than crRNA01 and crRNA03 (Supplementary Figure S1C).

To enhance the efficiency of the CRISPR/Cas12a system, the temperature, crRNA02 concentration, Cas12a concentration, and ssDNA reporter concentration were optimized. Real-time fluorescence collection was performed at various temperatures (Supplementary Figure S2A), showing better fluorescence intensity at 37 °C (Figure 3B), with clear identification of the PC and NTC groups under blue light instrument (Supplementary Figure S2B). An orthogonal design approach was employed to optimize the concentrations of crRNA02 and Cas12a (Supplementary Table S2), resulting in real-time fluorescence collection for nine groups (Supplementary Figure S3A). The optimized concentrations of crRNA02 and Cas12a were 150 nM and 50 nM, respectively (Figure 3C and Supplementary Table S2), with the visualization results of the nine groups displayed in Supplementary Figure S3B. Finally, it was found that ssDNA-FQ reporter exhibited similar real-time fluorescence variation curves at final concentrations of 200 and 250 nM (Supplementary Figure S4A), along with strong fluorescence intensity (Figure 3D) and clear visualization results (Supplementary Figure S4B). It was determined that the concentrations of 450 and 500 nM for the ssDNA-FB reporter were optimal, with the C line being prominent under negative conditions and the T line not showing any coloration (Figure 3E), while under positive conditions, the T line was prominent and the C line showed weak coloration (Figure 3F). Considering cost reduction and efficiency improvement, 200 nM ssDNA-FQ and 450 nM ssDNA-FB reporter were selected for subsequent experiments.

### 2.3 Specificity and sensitivity

**Figure 4:**
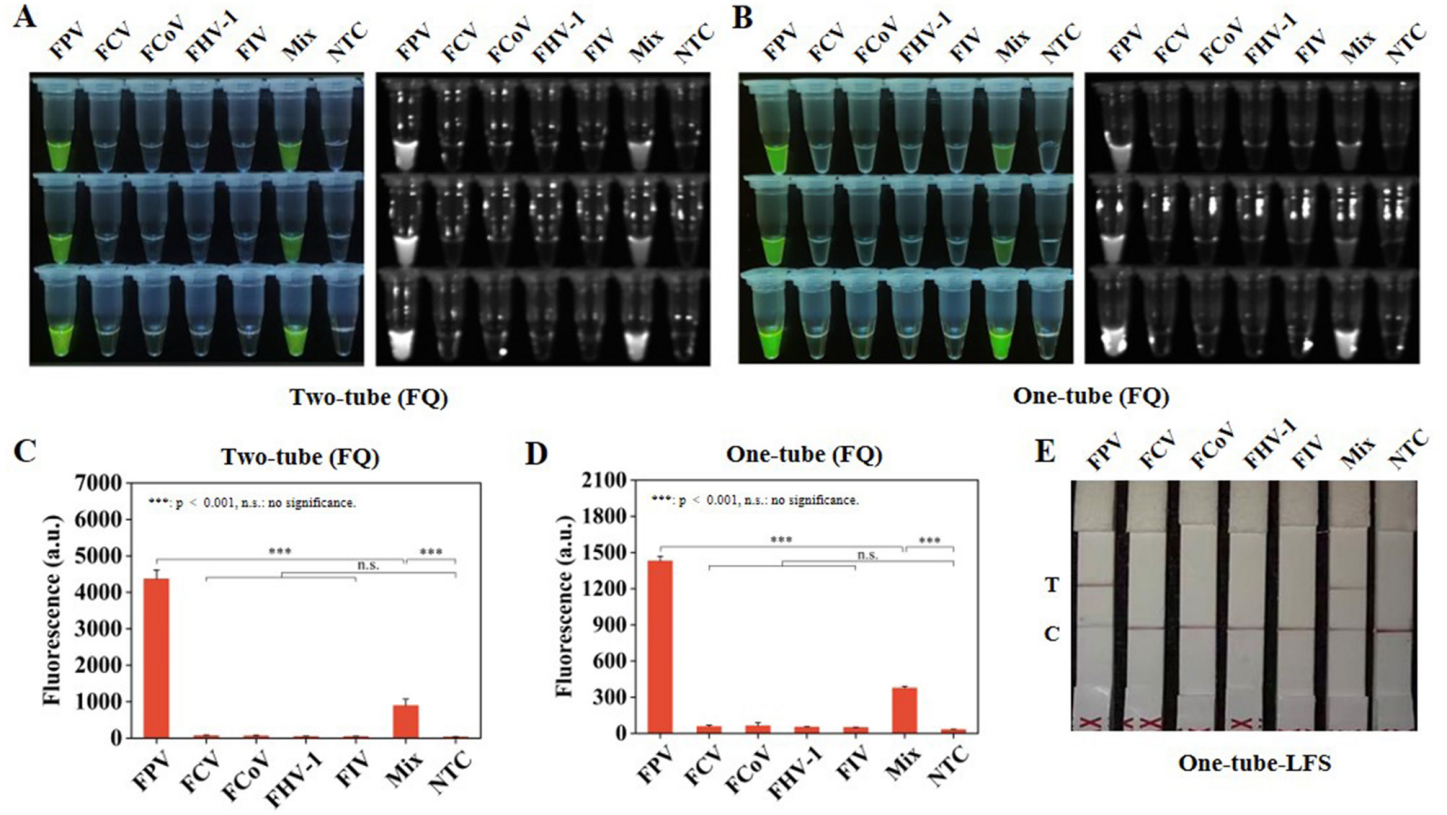
Specificity RAA-CRISPR/Cas12a system. **(A)** Visual fluorescence effect of specificity analysis by Two-tube method. **(B)** Visual fluorescence effect of specificity analysis by One-tube method. **(C)** Fluorescence intensity of specificity analysis at 30 min by Two-tube method. **(D)** Fluorescence intensity of specificity analysis at 30 min by One-tube method. **(E)** Specificity analysis by One-tube-LFS method. Error lines represent the mean ± S.D., of *n* = 3 replicates. ***p < 0.001. n. s., no significance.

Subsequently, the specificity of Two-tube, One-tube, and One-tube-LFS methods were evaluated. Significant visual fluorescence results were obtained under a blue light instrument for Two-tube and One-tube methods (Figures 4A, B). The real-time fluorescence curves (Supplementary Figures S5A, B) and fluorescence intensities at 30 min of both methods accurately distinguished FPV and Mix groups (Figures 4C, D). Additionally, in One-tube-LFS method, only the FPV and Mix groups showed color on the T line (Figure 4E), indicating positive results. No cross-reactivity occurred with other common feline infectious viruses for all three methods.

**Figure 5:**
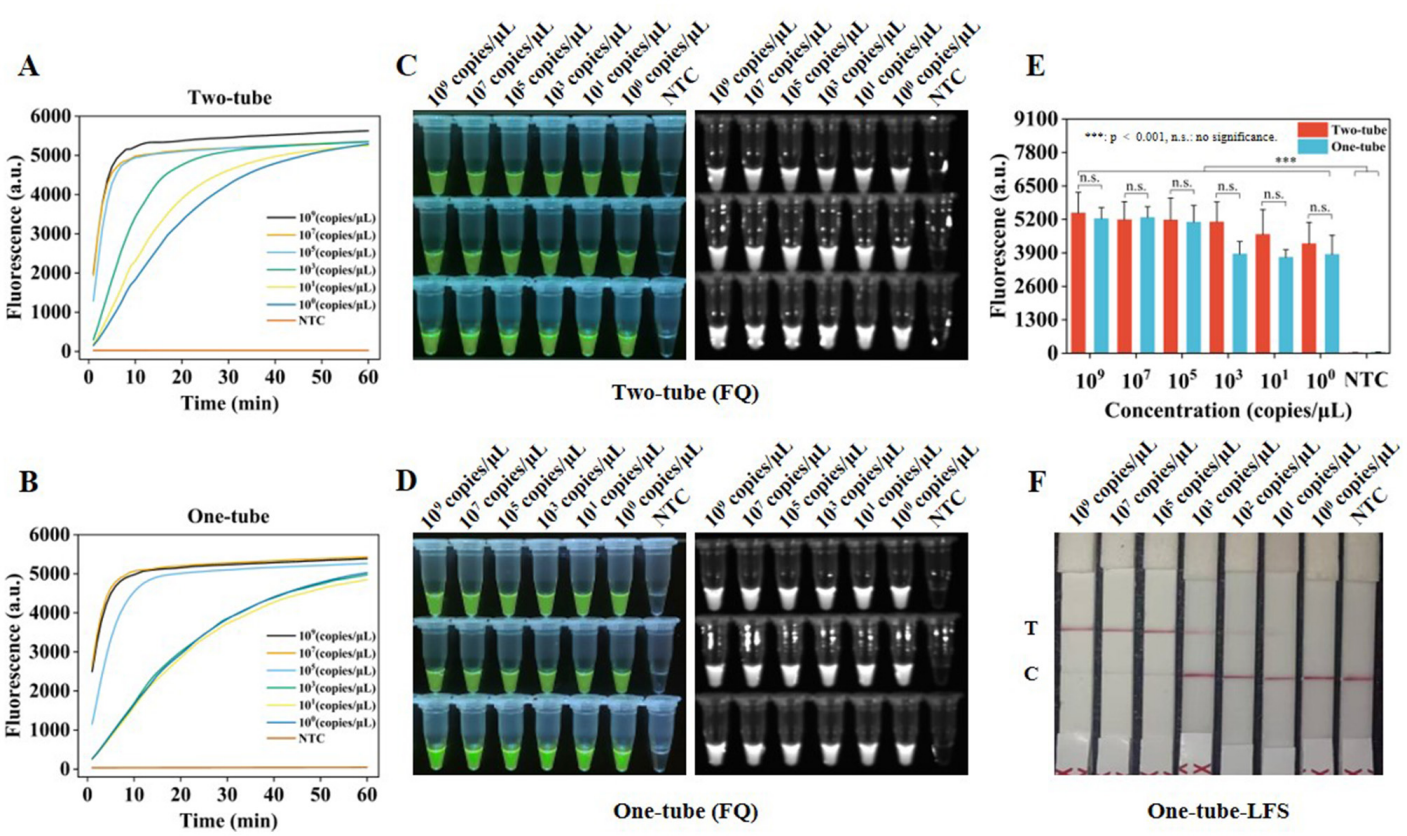
Sensitivity of RAA-CRISPR/Cas12a system. **(A)** Real-time fluorescence curves of sensitivity analysis by Two-tube method. **(B)** Real-time fluorescence curves of sensitivity analysis by One-tube method. **(C)** Visual fluorescence effect of sensitivity analysis by Two-tube method. **(D)** Visual fluorescence effect of sensitivity analysis by One-tube method. **(E)** Fluorescence intensity of sensitivity analysis at 30 min by Two-tube and One-tube methods. **(F)** Sensitivity analysis by One-tube-LFS method. Error lines represent the mean ± S.D., of *n* = 3 replicates. ***, *p* < 0.001. n. s., no significance.

To assess the sensitivity of Two-tube, One-tube, and One-tube-LFS methods, plasmid dilutions with concentrations ranging from 4.277 × 10^9^-4.277 × 10^0^ copies/μl were used as templates. According to the real-time fluorescence curves (Figures 5A, B) and fluorescence intensities at 30 min of reaction for Two-tube and One-tube methods, the LODs of both methods reached 4.277 × 10^0^ copies/μl (Figure 5E) and exhibited significant fluorescence effects (Figures 5C, D). Furthermore, the LOD of One-tube-LFS method reached 4.277 × 10^1^ copies/μl.

### 2.4 Detection of clinical samples

**Figure 6:**
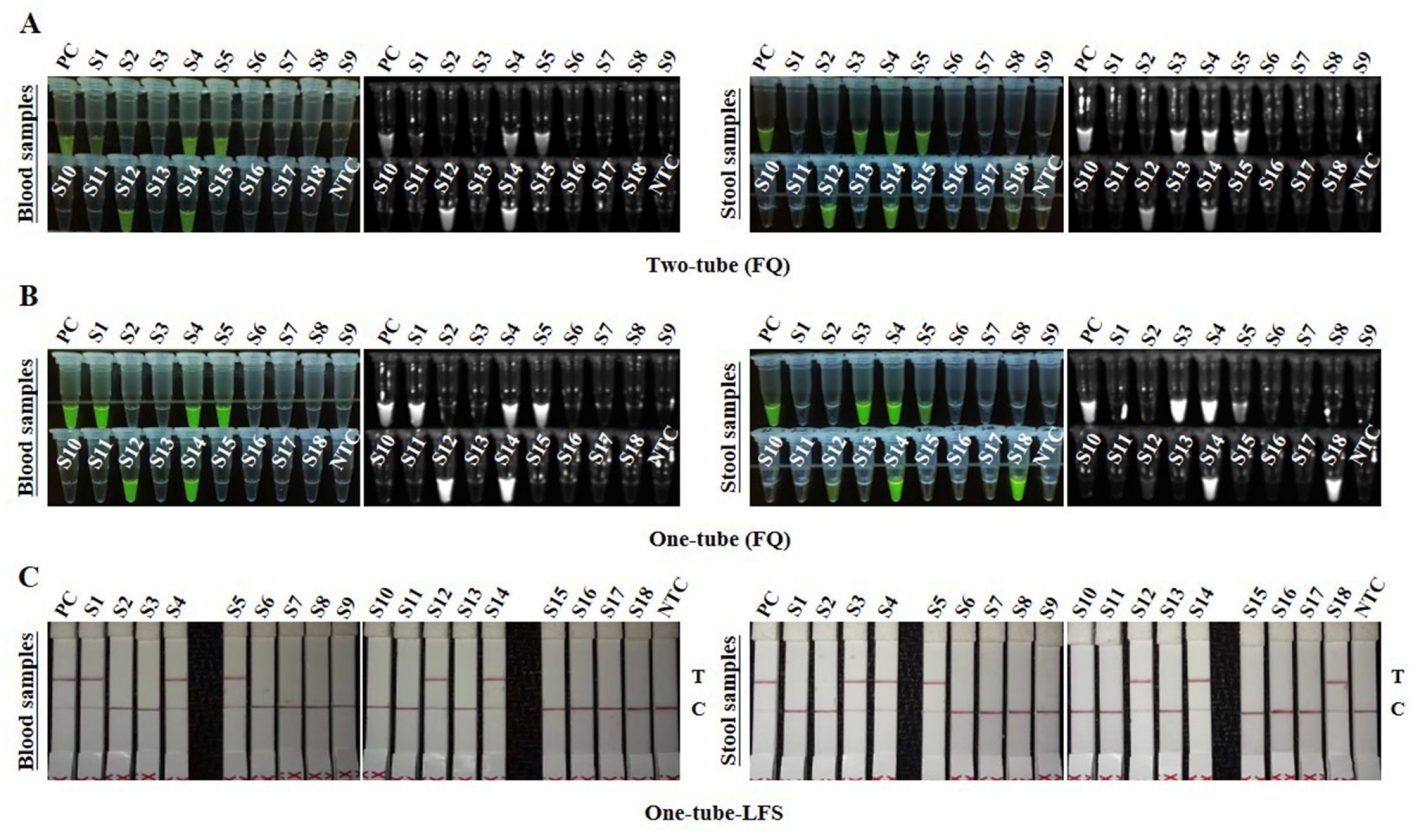
Detection of clinical samples with RAA-Cas12a system. **(A)** Detection results of Two-tube method. **(B)** Detection results of One-tube method. **(C)** Detection results of One-tube-LFS method.

A total of 36 clinical samples, including 18 blood samples and 18 fecal samples, were detected using Two-tube, One-tube, One-tube-LFS, and qPCR methods. In blood samples, five positive samples were detected by the three RAA-Cas12a methods (Figures 6A–C) and the qPCR method. In stool samples, six positive samples were identified by One-tube, One-tube-LFS, and qPCR methods (Figures 6B, C), while five positive samples were detected by Two-tube method (Figure 6A). As shown in Table 1, the consistency of Two-tube method with the qPCR method was 97.22%, whereas the consistencies of One-tube and One-tube-LFS methods with the qPCR method reached 100%.

**Table 1:** Clinical sample detection results of Two-tube, One-tube, One-tube-LFS, and qPCR methods.

**qPCR**	**Positive**	**Negative**	**Total**	**Consistency**
**Two-tube**				
Positive	10	0	10	97.22%
Negative	1	25	26	
Total	11	25	36	
**One-tube**				
Positive	11	0	11	100%
Negative	0	25	25	
Total	11	25	36	
**One-tube-LFS**				
Positive	11	0	11	100%
Negative	0	25	25	
Total	11	25	36	

## 3 Discussion

Rapid, accurate and sensitive detection of related pathogens was critical for providing appropriate treatment or biosecurity measures to prevent the spread of infectious diseases. However, traditional diagnostic methods required specialized laboratory equipment and were time-consuming and labor-intensive, failing to meet the current demands of POCT of infectious diseases. FPV was a highly pathogenic virus with a wide host range, particularly posing significant threats to felids. Many studies indicated that FPV had the potential for cross-species transmission (28–30). In this study, Two-tube, One-tube and One-tube-LFS methods were established based on the RAA-CRISPR/Cas12a system for the detection of FPV. The optimized RAA reaction temperature in the range of 36–40 °C yielded significant results (Figure 2C), which basically met the requirements for enzyme activity (31). Meanwhile, an amplification time of 30 min allowed for the accumulation of target molecules, facilitating subsequent detection. Additionally, the RAA reaction volume was reduced from the original 50–12.5 μl, which not only saved costs but also enhanced the interactions among reaction components (32).

To comprehensively evaluate the three methods, they were compared with other molecular methods for FPV detection (Table 2). Sensitivity and detection time were key indicators for evaluating the performance of nucleic acid detection. High sensitivity and rapid detection offered advantages for early identification of asymptomatic carriers or subclinical infections, effectively shortening the diagnosis and treatment cycle. In the RAA-CRISPR/Cas12a-FQ method, the LODs for Two-tube and One-tube methods were 4.277 copies/μl (Figure 5E), basically comparable to the sensitivity of similar RPA-Cas12a methods (1 copy/μl) (20). Additionally, the visual fluorescence results of the Two-tube and One-tube methods could be quickly presented by a blue light instrument in 5 min, and the fluorescence signals reached saturation as fast as 10 min (Figures 5A, B), with a detection time of 40 min, which was comparable to that of the RPA-Cas12a method (20). In the LFS method, the combination of multiple technologies increased the detection time of the One-tube-LFS method to 55 min, but the LOD was approximately 60% higher than that of the RPA-LFD method (10). The LOD of the One-tube-LFS method was 42.77 copies/μl (Figure 5F), comparable to the qPCR method (Supplementary Figure S6), slightly lower than the two-tube RPA-Cas12a-LFS method (2.1 copies/μl) (21) and the RPA-LFDA method (10 copies/μl) (33). The One-tube-LFS method could be visually observed in 3 min through commercial test strips, which was 10 min shorter than the detection time of the two-tube RPA-Cas12a-LFS method (65 min) (21) and more versatile than the RPA-LFDA method (which required preparing special test strips) (33). It reduced the contamination risk caused by opening the two-tube and saved costs, which was crucial for the rapid and accurate diagnosis of diseases. Compared with the gold-standard qPCR (34–38) and PCR (39, 40) methods for nucleic acid molecular detection, the Two-tube, One-tube and One-tube-LFS methods did not require precise and expensive temperature control equipment or cumbersome electrophoresis equipment. They only needed simple constant temperature instruments and blue light instruments to meet the on-site POCT needs. The detection sensitivity was comparable to or even higher than that of qPCR method, and the detection time was shortened from 2 h to within 1 h.

**Table 2:** Comparison of molecular detection methods for FPV.

**Methods**	**Instruments**	**Temperature**	**Detection time**	**LOD**	**Specificity**	**Results presentation**	**Ref**.
Two-tube	Mini dry bath	38 °C, 37 °C	40–60 min	4.277 copies/μl	FCV, FcoV, FHV-1, FIV	Blue light	This study
One-tube	Mini dry bath	38 °C, 37 °C	40–60 min	4.277 copies/μl	FCV, FCoV, FHV-1, FIV	Blue light	This study
One-tube-LFS	Mini dry bath	38 °C, 37 °C	55 min	42.77 copies/μl	FCV, FCoV, FHV-1, FIV	LFS	This study
RAA-Cas12a-LFS	Mini dry bath	37 °C	65 min	2.1 copies/μl	FCoV, FHV, FCV	LFS	(21)
RPA-Cas12a	Mini dry bath	37 °C	40 min	1 copies/μl	FIPV, FHV-1, FCV, MYC, CP	UV light	(20)
RPA-LFD	Mini dry bath	38 °C	35 min	100 copies/μl	FHV, FeCV, FIV	LFS	(10)
RPA-LFDA	Mini dry bath	39 °C	25 min	10 copies/μl	FCV, FHV-1	LFS	(33)
SEA	Mini dry bath	61 °C	40 min	6.6 pg/μ (3.43 × 10^6^ copies/μl)	FCoV, FCV, CDV, CCV	pH-sensitive dye	(45)
qPCR	qPCR instrument	60–95 °C	90 min	10 copies/μl	FCoV, FBoV-3, FeChPV, FBoV-2	Fluorescence curves	(38)
qPCR	qPCR instrument	54–95 °C	90 min	2.5 copies/μl	FIV, FeLV, FCoV, RV, PRV	Fluorescence curves	(34)
qPCR	qPCR instrument	60–95 °C	90 min	29.07 copies/μl	FAstV, FCV, FHV, FCoV	Fluorescence curves	(37)
qPCR-HRM	qPCR instrument	50–95 °C	100 min	4.2 copies/μl	FHV-1, FCV, CDV, CCV, CAV, CPIV	Fluorescence curves	(36)
qPCR	qPCR instrument	60–95 °C	90 min	47.4 copies/μl	FAstV, FBoV-1, FBoV-2, FBoV-3, FHV-1, FCV	Fluorescence curves	(35)
PCR	PCR and electrophoresis	55.6–98 °C	120 min	10^3^ copies/μl	FCoV, FAstV, FeKoV	Agarose gel	(39)
PCR	PCR and electrophoresis	51–95 °C	120 min	4.04 × 10^3^ copies/μl	FeKoV, FCV, FHV-1, FCoV, FIV, FeLV	Agarose gel	(40)
NanoPCR	PCR and electrophoresis	53–95 °C	120 min	2.64 × 10^4^ copies/μl	FCV, FHV-1, FIPV, RABV	Agarose gel	(44)
NanoPCR	PCR and electrophoresis	50–95 °C	120 min	7.97 × 10^2^ copies/μl	FHV, FCoV, FCV	Agarose gel	(43)

Studies have shown that gold nanoparticles can be closely bound to enzymes to inhibit non-specific amplification, and intermolecular interactions can be enhanced through charge effects (41, 42). When gold nanoparticles were introduced into the PCR system to establish NanoPCR, the sensitivity was increased by approximately 100 times compared with traditional PCR (43). The thermal conductivity of the PCR reaction mixture could be improved by the NanoPCR method, which greatly enhanced the detection sensitivity and shortened the reaction time. Three PCR false-negative samples were identified (44). Although the detection sensitivity and time of the NanoPCR method were not as ideal as the three methods in this study, integrating nanomaterials into the nucleic acid detection system can improve the sensitivity and amplification efficiency of FPV detection while inhibiting non-specific reactions, providing a promising approach for the subsequent optimization of the three POCT methods in this study.

Furthermore, the three methods in this study could specifically detect FPV without cross-reacting with other feline pathogens such as FCV, FCoV, FHV-1, and FIV (Figure 4), consistent with the specificity of the methods in Table 2, demonstrating their good specificity. In the detection of clinical samples, compared with the gold-standard qPCR method (Table 1), the consistency between the Two-tube method and qPCR reached 97.22%, while the One-tube and one-tube-LFS methods both showed 100% consistency with qPCR, highlighting their practical application value. Therefore, the three methods developed in this study exhibit good advantages, which will provide a guarantee for the early screening and diagnosis of FPV in resource-limited environments.

## 4 Conclusion

In this study, Two-tube, One-tube, and One-tube-LFS methods were developed based on the RAA-CRISPR/Cas12a system. The RAA reaction and CRISPR/Cas12a reaction were carried out sequentially without interference, resolving the issues of reduced DNA amplification efficiency and sensitivity decline in traditional integrated RAA-Cas12a methods and significantly enhancing the detection performance of RAA-Cas12a. Simultaneously, one-tube design addressed the aerosol contamination problem caused by open-tube operations. All three methods enabled visual detection. After nucleic acid amplification, Two-tube and One-tube methods allowed fluorescence results to be observed using a blue light instrument at 37 °C in 5 min (LOD was 4.277 copies/μl), while One-tube-LFS method provided visible positive results in 3 min (LOD was 42.77 copies/μl). The high sensitivity, specificity, and rapid accuracy of these RAA-CRISPR/Cas12a methods indicate their suitability for FPV detection in resource-limited and clinical settings, offering methodological references for the early rapid diagnosis, epidemiological investigation, and epidemic prevention and control of FPV.

## 5 The experimental section

### 5.1 Materials

All primers and crRNA were synthesized by Sangon Biotech Co., Ltd (Shanghai, China) and the sequence information was listed in Supplementary Table S1. The LbCas12a (Cpf1) and Reaction Buffer 1 were provided by MAGIGEN Biotechnology Co., Ltd (Guangzhou, China). The single stranded DNA–fluorescently quenched (ssDNA-FQ) reporter was provided by EZassay Biotechnology Co., Ltd (Shenzhen, China). The ssDNA fluorescein biotin (ssDNA-FB) reporter was provided by Baoying Tonghue Biotechnology Co., Ltd (Beijing, China), and the CRISPR test strip (TS104) was provided by Synda Genetic Technology Co., Ltd (Suzhou, China). The DNA Rapid Thermostatic Amplification Kit (basic) was purchased from Amp-Future Biotech Co., Ltd (Changzhou, China). DH5α competent cells and the rapid plasmid extraction kit were provided by TIANGEN Biotech Co., Ltd (Beijing, China). The 6 × DNA loading buffer, 10,000 × YeaRed nucleic acid dye, and DL 2000 Marker were purchased from Yeasen Biotech Co., Ltd (Shanghai, China).

Plasmid standards for FCV (Feline calicivirus), FCoV (Feline coronavirus), FHV-1 (Feline herpesvirus type 1), and FIV (Feline immunodeficiency virus) were provided by YANYUAN Biotechnology Co., Ltd (Shenzhen, China). The FPV clinical samples, which consisted of 18 blood samples and 18 stool samples, were from the local pet hospital.

### 5.2 Primer and crRNA

According to the GenBank (https://www.ncbi.nlm.nih.gov/genbank/) had released FPV VP2 gene sequences (registration number: KC473946.1), two pairs of RAA primers were designed using Primer Premier 5.0 software, and crRNA was designed by EZASSAY (https://ezassay.com/rna). They were synthesized by Sangon Biotech Co., Ltd (Shanghai, China), including one pair of qPCR primers from Academy of Animal Science and Veterinary Sciences (Jilin, China).

### 5.3 The positive recombinant plasmid pUC57-VP2

The standard plasmid template of FPV was designed and synthesized by Sangon Biotech Co., Ltd. (Shanghai, China), and inserted into the pUC57 vector to construct the recombinant plasmid pUC57-VP2. Subsequently, pUC57-VP2 was transformed into DH5α competent cells and coated in LB solid medium containing ampicillin, followed by incubation overnight at 37 °C. Monoclonal bacteria were selected and cultured in LB liquid medium with ampicillin at 37 °C, and the resulting culture was processed by a rapid plasmid extraction kit (centrifuge column type) to extract the plasmid, yielding the FPV recombinant plasmid. Finally, the concentration of the recombinant plasmid was determined by a NanoOne micro-spectrophotometer from Youning Instruments Co., Ltd. (Hangzhou, China). The copy number (***CN***) of the plasmid was calculated according to Equation 1, 2. The calculated copy number of the FPV plasmid was 4.277 × 10^11^ copies/μl. The standard plasmid was subsequently diluted from 4.277 × 10^11^ copies/μl to 4.277 × 10^0^ copies/μl and stored at −20 °C for later use.


$$\begin{array}{l} CN=\frac{C\times 10^{-9}\times N_{A}}{M_{W}} \end{array}$$ (1)

$$\begin{array}{l} M_{W}=L\times Da \end{array}$$ (2)

In the formula, ***CN*** is plasmid copy number (copies/μl), ***C*** is plasmid concentration (ng/μl), ***N***_***A***_ is Avogadro constant (copies/mol), ***M***_***W***_ is molecular weight (g/mol), ***L*** is sequence length (bp) and ***Da*** is average molecular weight (dalton/bp).

### 5.4 RAA system

The 25 μl RAA reaction mixture was prepared. First, 14.7 μl of buffer was completely dissolved and mixed with lyophilized powder. Then, 1 μl each of 10 μM upstream and downstream primers, 1.3 μl of B buffer, 2.5 μl of plasmid template, and 4.5 μl of sterile enzyme water were added. The mixture was incubated at 38 °C for 30 min in a G100 dry bath incubator made by Youning Instruments Co., Ltd. (Hangzhou, China). After the reaction, the amplification products were extracted with an equal volume of Tris-saturated phenol/chloroform/isoamyl alcohol (25:24:1) to reduce protein interference in electrophoresis. The extracted products were verified by 2% agarose gel electrophoresis. Finally, the electrophoresis results were observed and recorded using a GelView 5000Plus intelligent gel imaging system from Boluteng Biotechnology Co., Ltd. (Guangzhou, China).

### 5.5 Two-tube method

The Two-tube RAA-CRISPR/Cas12a reaction with a volume of 40 μl consisted of 12.5 μl RAA system and 27.5 μl CRISPR/Cas12a system. First, the 12.5 μl RAA system was amplified in a PCR tube for 30 min. The RAA products were then mixed with 27.5 μl CRISPR/Cas12a system, and real-time fluorescence detection was immediately performed. The 27.5 μl CRISPR/Cas12a system included 50 nM Cas12a, 150 nM crRNA02, 200 nM ssDNA-FQ reporter, 1 × reaction buffer 1, and enzyme-free sterile water. Fluorescence signals were collected for 30 min at 37 °C using a SLAN-96S fully automatic medical PCR analysis system (Hongshi Medical Technology Co., Ltd., Shanghai, China), with readings every minute. After collection, the fluorescence effect was observed under blue light, and images were recorded.

### 5.6 One-tube method

The One-tube RAA-CRISPR/Cas12a reaction system of 40 μl was prepared. 12.5 μl of RAA system and 27.5 μl of CRISPR/Cas12a system were placed at the bottom and cap of a PCR tube, respectively. The tube was transferred to a fluorescence monitor for 30-min amplification. After amplification, RAA products were mixed with 27.5 μl CRISPR/Cas12a reagent. Real-time fluorescence detection was conducted immediately. After fluorescence collection, the effect was observed under blue light, and images were recorded.

### 5.7 One-tube-LFS method

Referring to One-tube method, the amplified RAA products were mixed with 27.5 μl of the CRISPR/Cas12a system and incubated at 37 °C for 25 min. Contrast to One-tube method, the 200 nM ssDNA-FQ was replaced with 450 nM ssDNA-FB in the 27.5 μl of CRISPR/Cas12a system. After incubation, the reaction product was mixed with an equal volume of sterile enzyme-free water, and 40 μl of the mixture was dropped onto the sample pad of the LFS. Finally, the detection results were observed by naked eyes and recorded. In the LFS, the conjugate pad was pre-embedded with colloidal gold-labeled mouse anti-FAM antibodies, which could bind to both the FAM end of ssDNA-FB and the anti-mouse antibodies coated on the T line. Meanwhile, streptavidin immobilized on the C line could bind to the Biotin end of ssDNA-FB.

### 5.8 Specificity and sensitivity

To evaluate the specificity of the three methods based on the RAA-CRISPR/Cas12a system, standard plasmid samples of FPV, FCV, FCoV, FHV-1, and FIV at a concentration of 10 ng/μl were used for detection. Meanwhile, a mixture (Mix) composed of equal proportions of the above plasmid standards was employed as a control. Enzyme-free sterile water was used as a blank control. Additionally, FPV standard samples at different concentrations (4.277 × 10^9^-4.277 × 10^0^ copies/μl) were used to assess sensitivity, with enzyme-free sterile water serving as a blank control. Each sample group was prepared in triplicate, and each sample was subjected to three independent experiments.

### 5.9 Clinical sample testing

FPV clinical samples were extracted by DNA extraction kit and then detected. Specifically, 200 μl of the pretreatment sample, 20 μl of Proteinase K, 250 μl of lysis buffer RLC, and 250 μl of isopropanol were added to a centrifuge tube and mixed by vortexing. The mixture was incubated at 56 °C for 5 min and then cooled to room temperature. Subsequently, the mixture was transferred to an adsorption column and centrifuged at 12,000 rpm for 1 min, discarding the waste liquid in the collection tube. Next, 700 μl of wash buffer PWT was added, followed by centrifugation at 12,000 rpm for 1 min, with the waste liquid discarded, and repeated once. The adsorption column was centrifuged at 12,000 rpm for 2 min to remove the waste liquid, then placed in a new centrifuge tube with the lid open at room temperature for 1 min. Afterward, 100 μl of elution buffer was added, the lid was closed, and the mixture was left at room temperature for 2 min. Finally, the viral DNA obtained from the adsorption column was used as a template for amplification and stored at −20 °C for later use.

Subsequently, the three methods were applied to detect 18 blood samples and 18 stool samples, including no-template controls and positive controls. Additionally, SYBR Green I based qPCR assay was performed on these clinical samples to validate the accuracy of the three methods of the RAA-CRISPR/Cas12a system. In brief, 10 μl of 2 × Taq Pro Universal SYBR qPCR Master Mix, 0.4 μl of 10 μM upstream and downstream primers, 1.5 μl of template, and 7.7 μl of sterile enzyme-free water were added to a 200 μl PCR tube for a total of 20 μl of qPCR reaction mixture. The reaction mixture was detected by qPCR detection on the SLAN-96S PCR instrument, following a three-step qPCR protocol. First, the mixture was pre-denatured at 95 °C for 30 s. Then, the mixture was followed by 40 cycles of denaturation at 95 °C for 10 s, annealing at 54 °C for 30 s, and extension at 72 °C for 30 s. Finally, a terminal extension at 72 °C for 10 min.

### 5.10 Statistical analysis

Data generation and collection were carried out with SLAN-96S PCR instrument. Data management, analysis, and graphics generation were performed using Microsoft Excel 2016 (Microsoft, USA) and OriginPro 2024b software (Origin Lab, Northampton, MA, USA). All statistical analyses were performed using a one-way analysis of variance with Tukey's comparison test. Specifically, all experimental groups were compared against the NTC group to determine the statistical significance of differences, and the *p*-values presented in the figures were derived from these specific comparisons. Data were presented as mean ± standard deviation (S.D.; *n* = 3). A *p*-value less than 0.05 was considered as statistical significance.

## Data Availability

The original contributions presented in the study are included in the article/Supplementary material, further inquiries can be directed to the corresponding authors.
