# Supplementary material for: RAA-CRISPR/Cas12a-driven two-tube, one-tube and one-tube-LFS for rapid detection of feline parvovirus
Source: Front Vet Sci. 2025 Dec 12;12:1707332. doi: 10.3389/fvets.2025.1707332 (PMC12741964; doi:10.3389/fvets.2025.1707332)
Supplement: Supplementary file 1 [file Table_1.docx]

**Supplementary Information**

**Tables and Figures**

**Table S1**. The sequence information of primers and crRNA.

**Table S2**. The concentrations of crRNA02 and Cas12a incorporated in the nine different complexes.

**Figure S1**. Optimization of crRNA.

**Figure S2**. Optimization of temperature.

**Figure S3**. Optimization of concentration of crRNA and Cas12a.

**Figure S4**. Optimization of concentration of ssDNA-FQ reporter.

**Figure S5**. Specificity of RAA-Cas12a system.

**Figure S6.** Performance of the qPCR method.

**Table S1**. The sequence information of primers and crRNA.

| Name | Sequence (5’ – 3’) | Sequence Size (bp) | Product Size (bp) |
| --- | --- | --- | --- |
| RAA-F1 | TCAACCTGCTGTCAGAAATGAAAGAGCTAC | 30 | 194 |
| RAA-R1 | TTTCTGGCATATTTAAATGTACAAGTCTGC | 30 |  |
| RAA-F2 | ATAATAATGATTTAACTGCATCATTGATGG | 30 | 159 |
| RAA-R2 | ATGTCCTATCCCATTGAAAATAATATCTCC | 30 |  |
| qPCR-F | AATGCTTGGGGAGTTTGGTTT | 21 | 147 |
| qPCR-R | TTTAGTTGGTGGCTGAGTAGCAGA | 24 |  |
| crRNA01 | CACCCAUCCGUUUUCCAAAAAUUAUCUACAACAGUAGAAAU | 41 |  |
| crRNA02 | AAUUCCGUCUGAUUAUUGAAAGUAUCUACAACAGUAGAAAU | 41 |  |

**Table S2**. The concentrations of crRNA02 and Cas12a incorporated in the nine different complexes.

| Group | crRNA02 | Cas12a |
| --- | --- | --- |
| 1 | 50 nM | 50 nM |
| 2 | 50 nM | 100 nM |
| 3 | 50 nM | 150 nM |
| 4 | 100 nM | 50 nM |
| 5 | 100 nM | 100 nM |
| 6 | 100 nM | 150 nM |
| 7 | 150 nM | 50 nM |
| 8 | 150 nM | 100 nM |
| 9 | 150 nM | 150 nM |


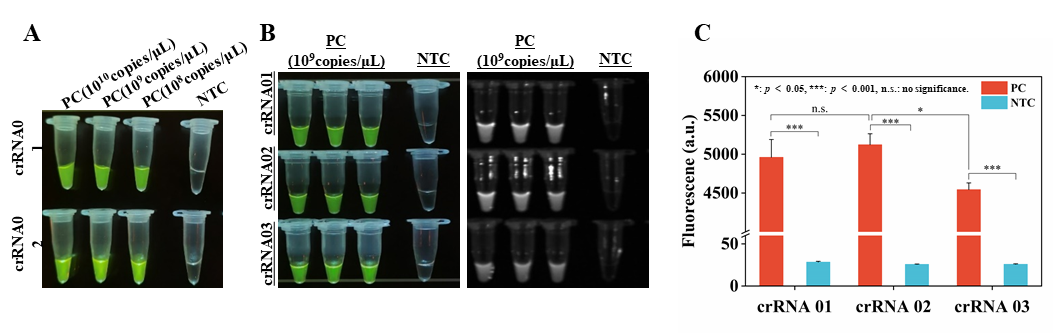


**Figure S1**. Optimization of crRNA. (A) Validation of the crRNA sequence. (B) Visual fluorescence effect of crRNA in the three groups. (C) Fluorescence intensity of three crRNAs at 30 min. Error lines represent the mean ± S.D., of n = 3 replicates. *, P ＜ 0.05. **, P ＜ 0.01. ***, P ＜ 0.001. n. s., no significance.


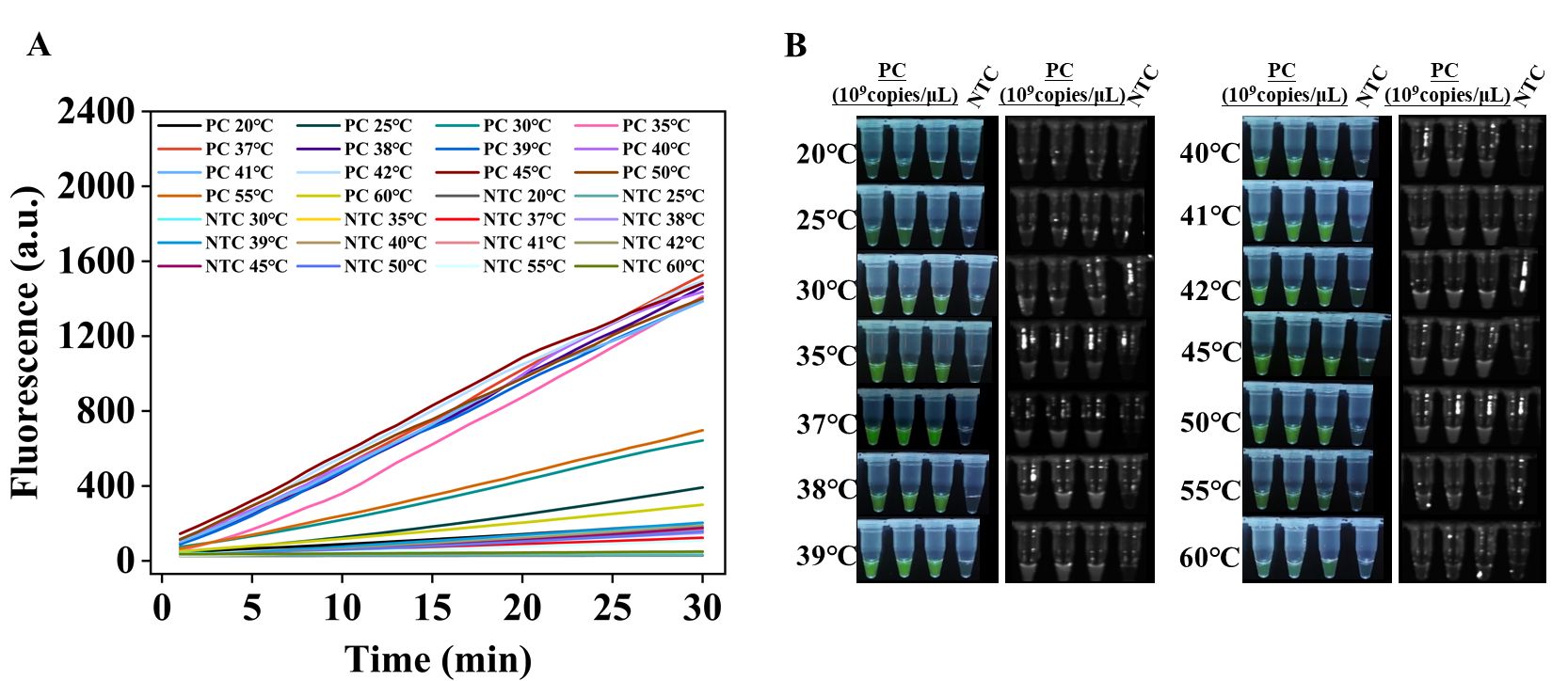


**Figure S2**. Optimization of temperature. (A) Real-time fluorescence acquisition curves at different temperatures. (B) Visual fluorescence effect at different temperatures.


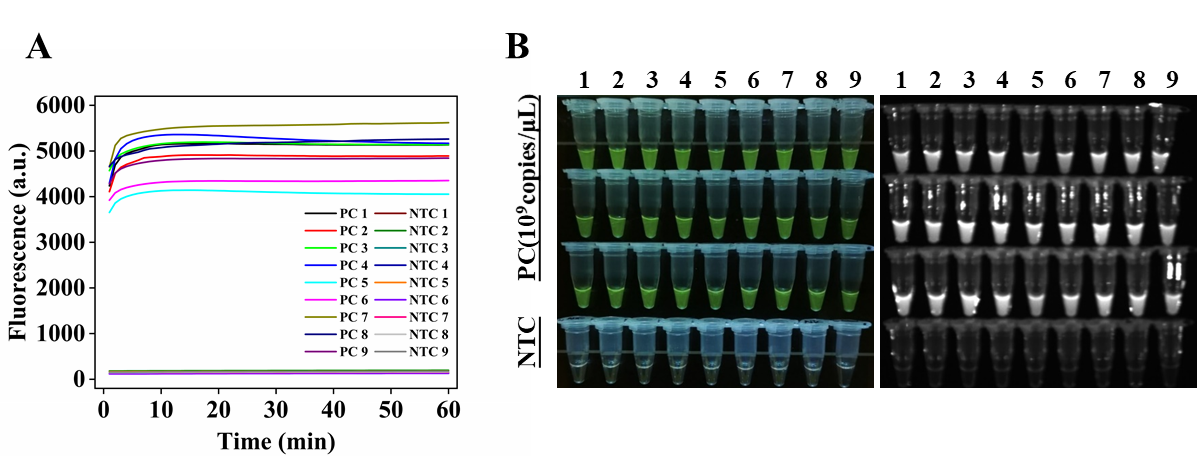


**Figure S3**. Optimization of concentration of crRNA and Cas12a. (A) Real-time fluorescence acquisition curves in the nine groups of complexes. (B) Visual fluorescence effect of different groups.


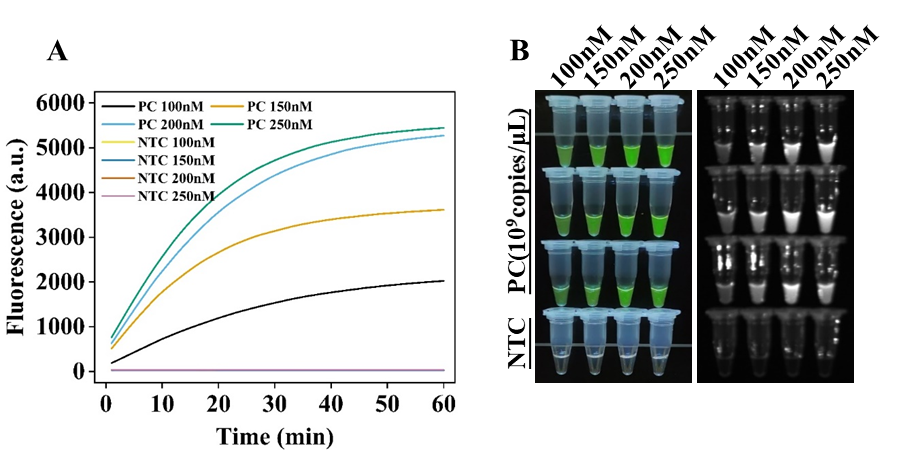


**Figure S4**. Optimization of concentration of ssDNA-FQ reporter. (A) Real-time fluorescence collection curves of ssDNA-FQ reporter. (B) Visual fluorescence effect of ssDNA-FQ reporter.


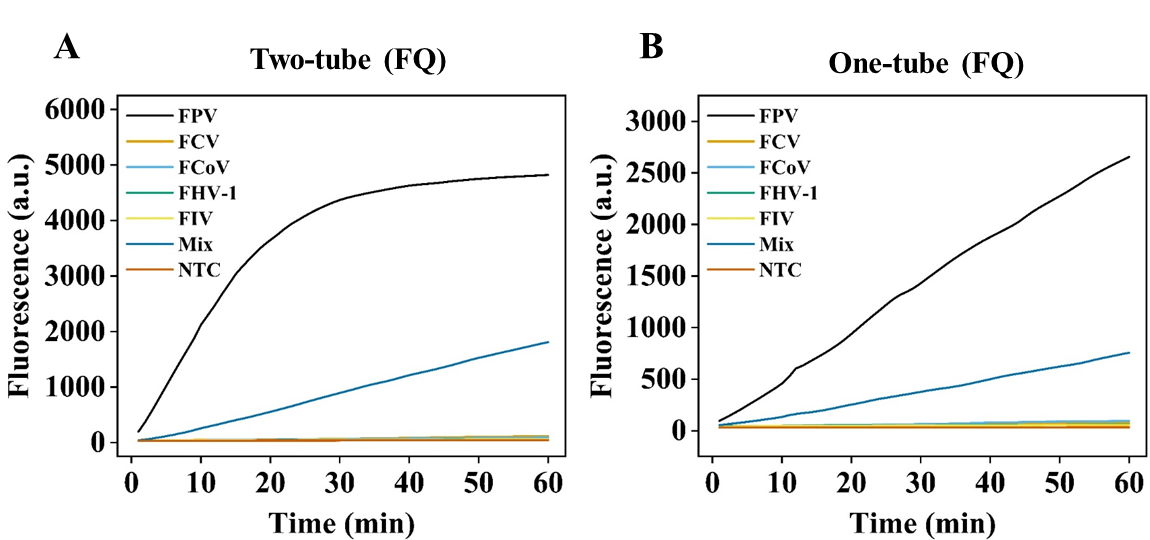


**Figure S5**. Specificity of RAA-Cas12a system. (A) Real-time fluorescence collection curves of specificity analysis by Two-tube method. (B) Real-time fluorescence collection curves of specificity analysis by One-tube method.


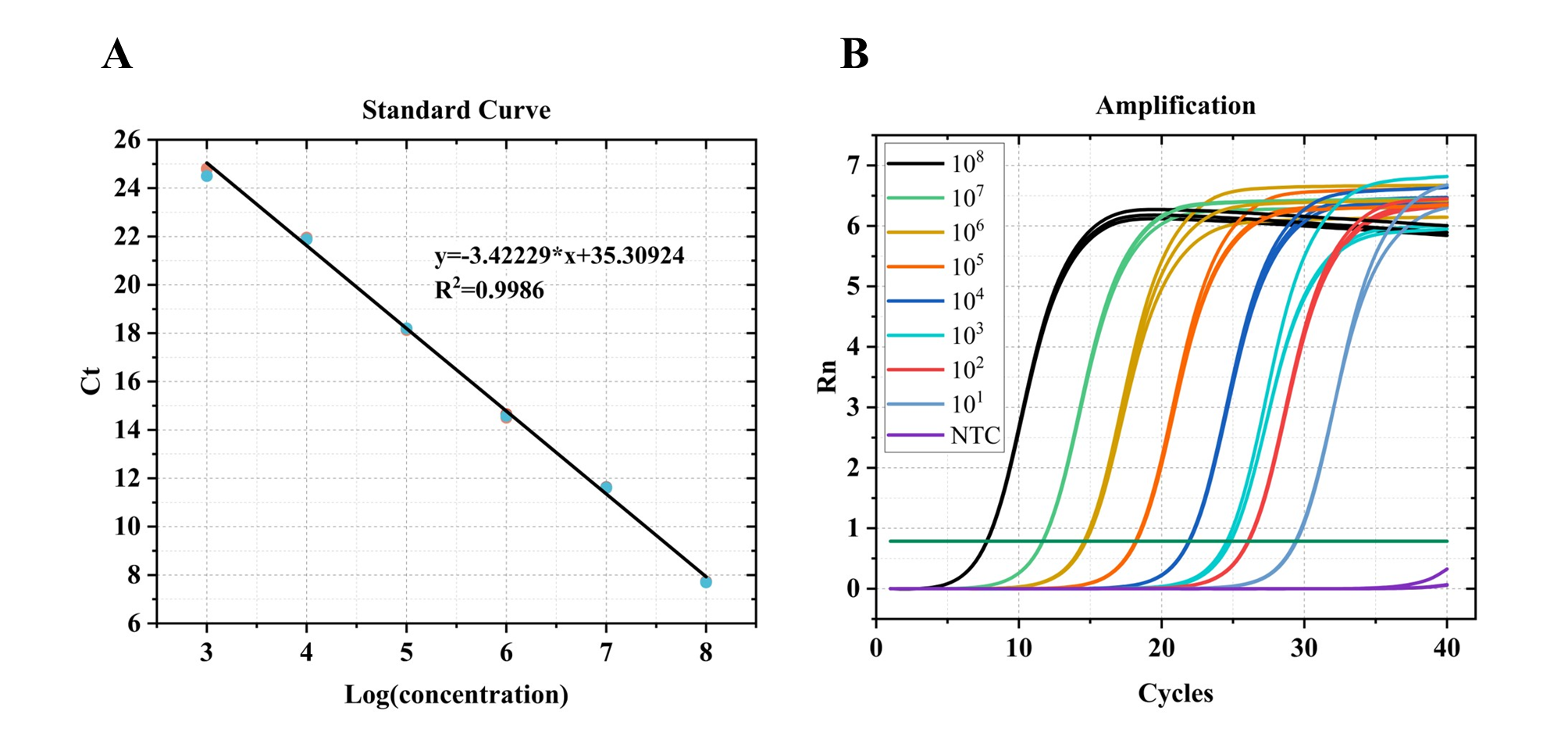


**Figure S6**. Performance of the qPCR method. (A) Standard curve of the qPCR method. (B) Sensitivity of the qPCR method.
